# Hemophagocytic Syndrome Associated with *Mycoplasma pneumoniae* Pneumonia

**DOI:** 10.1155/2013/586705

**Published:** 2013-07-18

**Authors:** Yuji Koike, Nao Aoki

**Affiliations:** Department of Pediatrics, Disaster Medical Center, 3256 Midori-cho, Tachikawa, Tokyo 190-0014, Japan

## Abstract

*Mycoplasma pneumoniae* (Mp) sometimes causes immunological complications in children. We present a rare case of hemophagocytic syndrome (HPS) caused by Mp in a previously healthy 7-year-old Japanese girl. A chest radiograph obtained to evaluate the source of her fever showed infiltration in the lower right lung with mild splenomegaly. We could diagnose the patient with HPS on the basis of the hemophagocytic-lymphohistiocytosis- (HLH) 2004 criteria. She met the criteria for fever, splenomegaly, neutrophil count (<1,000/**μ**L), platelet count (<10.0 × 10^4^/**μ**L), fasting triglyceride level (>265 mg/dL), and ferritin level (>500 ng/mL). Furthermore, a peripheral blood smear showed an increased number of monocytes/macrophages with erythrophagocytosis. Treatment with clarithromycin and prednisolone, which was initiated soon after the diagnosis, was successful. Mp infection might partly progress to HPS in certain conditions. Clinicians should be aware of HPS caused by Mp and start appropriate treatment as soon as possible if the disease is suspected.

## 1. Introduction

*Mycoplasma pneumoniae* (Mp) is well known to cause upper and lower respiratory tracts infections, including pharyngitis, bronchitis, and pneumonia. Mp also causes immunological complications in children, such as Stevens-Johnson syndrome, Guillain-Barré syndrome, Kawasaki disease [1], aseptic meningitis, and hemophagocytic syndrome (HPS). HPS is characterized by abnormal activation of monocytes/macrophages and consequently overproduction of proinflammatory cytokines [2, 3]. Among various types of HPS, secondary HPS is often associated with infections such as virus, bacteria, fungi, and parasites [4]. However, Mp rarely causes secondary HPS, and there have so far been few reports concerning HPS due to Mp [5, 6]. We herein report a 7-year-old girl who was diagnosed as having HPS associated with Mp pneumonia, and we successfully treated her with an appropriate antibiotic along with corticosteroid soon after the diagnosis.

## 2. Case Presentation

**Figure 1:**
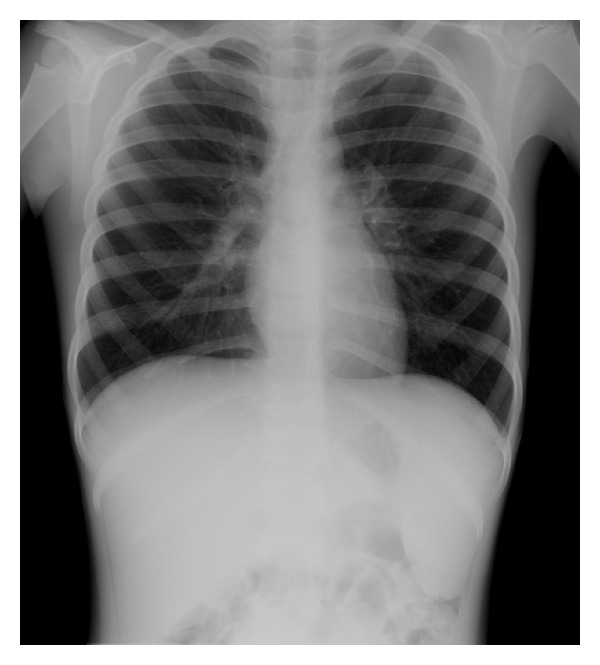
A chest radiograph of the patient showing infiltration in the lower right lung and mild splenomegaly.

**Figure 2:**
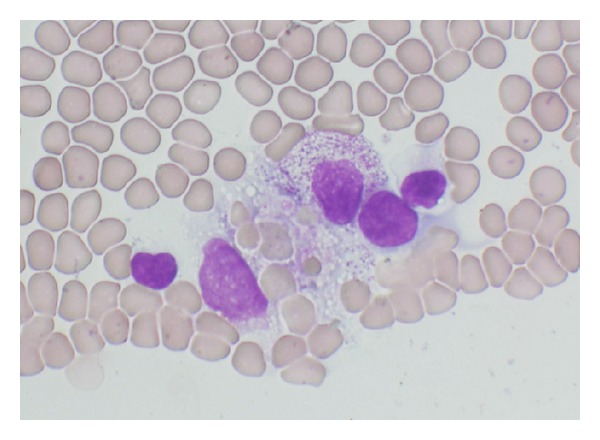
A peripheral blood smear of the patient (May-Giemsa, 400x) showing an increased number of monocytes/macrophages with erythrophagocytosis.

A previously healthy 7-year-old Japanese girl presented to her physician with three-day history of fever, cough, and malaise and was referred to our outpatient clinic by the physician for evaluation of fever and leukocytopenia. Physical examinations revealed mild hepatosplenomegaly, however, no lymphadenopathy nor skin rash. Chest auscultation revealed also normal breath sounds. A chest radiograph obtained to evaluate the source of her fever showed infiltration in the lower right lung with mild splenomegaly (Figure 1). Laboratory findings were as follows: white blood cell (WBC) counts 1,200 /*μ*L with 45% neutrophils, 34% lymphocytes, and 20% monocytes, red blood cell (RBC) counts 425 × 10^4^ /*μ*L, hemoglobin (Hb) 11.8 g/dL, hematocrit (Ht) 33.3%, platelet (Plt) counts 3.7 × 10^4^ /*μ*L, aspartate aminotransferase (AST) 48 IU/L, alanine aminotransferase (ALT) 28 IU/L, lactate dehydrogenase (LDH) 921 IU/L, triglyceride (TG) 193 mg/dL, C-reactive protein (CRP) 6.27 mg/dL, ferritin 1565.0 ng/mL, immunoglobulin (Ig) G 1,350 mg/dL, IgA 212 mg/dL, IgM 98 mg/dL, the erythrocyte sedimentation rate (ESR) 102 mm/hour, and antibody titer for Mp (phytohemagglutinin, PHA) 2,560×. Serological tests for Epstein-Barr virus, cytomegalovirus, and adenovirus were negative throughout the course. A peripheral blood smear showed an increased number of monocytes/macrophages with erythrophagocytosis (Figure 2). Therefore, we diagnosed the case to have HPS associated with pneumonia caused by Mp infection. After the diagnosis, she admitted to the hospital (day 4 of the disease) and was treated with 200 mg/day of clarithromycin and 40 mg/day of prednisolone (2 mg/kg) orally. The fever did not soon subside; however, the abnormal laboratory findings were gradually improved since the nadir of pancytopenia (WBC counts 800 /*μ*L, Hb 10.7 g/dL, and Plt counts 3.2 × 10^4^ /*μ*L) on day 5, and so, we did not perform a bone marrow examination. Her fever resolved on day 6, and the corticosteroid therapy was gradually tapered. She was discharged from the hospital on day 11 and has been well for more than three years.

## 3. Discussion

HPS induced by Mp infection in children is rare [5, 6]. In our case, we could diagnose her to have HPS by HLH-2004: diagnostic and therapeutic guidelines for hemophagocytic lymphohistiocytosis [4]. She met the criteria of fever, splenomegaly, neutrophils <1,000 /*μ*L and Plt counts <10.0 × 10^4^ /*μ*L, fasting TG >265 mg/dL (maximum level of TG was 434 mg/dL on day 10), hemophagocytosis, and ferritin >500 ng/mL (maximum level of ferritin was 2,251 ng/mL on day 10). Consequently, we immediately treated her with clarithromycin along with prednisolone. High levels of serum TG and ferritin are considered to be correlated with high tumor necrosis factor (TNF)-*α*, one of the proinflammatory cytokines [3, 7]. As well, Oishi et al. reported that serum levels of LDH and interleukin (IL)-18 in children with Mp pneumonia were significantly correlated [8]. We could not examine the exact cytokine profiles, as her maximum level of LDH was 1,384 IU/L on day 8; we consider that hypercytokinemia existed in our case.

Mp infection is thought to be self-limiting in nature and responds well to antimicrobial therapy. Recently, refractory Mp pneumonia, defined as clinical and radiological deterioration despite appropriate antibiotic therapy for more than one week, was increasingly reported [9]. Refractory Mp infection is also suggested to be complicated by hypercytokinemia. Therefore, combination therapy with macroides and corticosteroids is suggested to be effective for refractory Mp infections, probably because the therapy could decrease cytokines and chemokines produced by Mp infections [6, 10]. The immunopathological differences between refractory Mp infection and HPS are not fully elucidated [11]; however, we speculate that, without the therapy, refractory Mp infection might partly progress to HPS in certain conditions. Considering the fact that almost HPS is relatively severe, the intervention should be done soon after the diagnosis or at a stage prior to HPS, if possible [4]. 

Therefore, we suggest a therapeutic strategy for Mp infection later. When Mp infection is strongly suspected, an appropriate antibiotic such as macroides with enough doses is administered. In addition to the serum levels of LDH >500 IU/L and/or ferritin >150 ng/mL, if the patient does not recover or the fever subsides over three days after the antibiotic therapy, corticosteroid therapy (e.g., using prednisolone 2 mg/kg/day for 3–5 days) should be started [10]. 

In conclusion, clinicians should be aware of HPS induced by Mp infection and should treat the disease appropriately as soon as possible when it is suspected.
